# The impact of colectomy on the risk of cardiovascular disease among patients without colorectal cancer

**DOI:** 10.1038/s41598-020-59640-w

**Published:** 2020-02-19

**Authors:** Chin-Chia Wu, Ta-Wen Hsu, Chia-Chou Yeh, Cheng-Hung Lee, Mei-Chen Lin, Chun-Ming Chang

**Affiliations:** 1 Division of Colorectal Surgery, Dalin Tzu Chi Hospital, Buddhist Tzu Chi Medical Foundation, Chiayi, Taiwan; 2 Department of Chinese Medicine, Dalin Tzu Chi Hospital, Buddhist Tzu Chi Medical Foundation, Chiayi, Taiwan; 3 Department of General Surgery, Dalin Tzu Chi Hospital, Buddhist Tzu Chi Medical Foundation, Chiayi, Taiwan; 4 Management Office for Health Data, China Medical University Hospital, Taichung, Taiwan; 5 Department of General Surgery, Hualien Tzu Chi Hospital, Buddhist Tzu Chi Medical Foundation, Hualien, Taiwan; 6 College of Medicine, Tzu Chi University, Hualien, Taiwan; 7 School of Post-Baccalaureate Chinese Medicine, Tzu Chi University, Hualien, Taiwan; 8 College of Medicine, China Medical University, Taichung, Taiwan

**Keywords:** Cardiovascular diseases, Epidemiology

## Abstract

Cardiometabolic disorders were discussed and might be changed by microbiota in recent years. Since the colon acts as the primary reservoir of microbiota, we designed the present study to explore the association between colectomy and cardiovascular disease (CVD). We identified a total of 18,424 patients who underwent colectomy between 2000–2012 for reasons other than colorectal cancer from the National Health Insurance Research Database of Taiwan. Patients were matched with 18,424 patients without colectomy using a 1:1 propensity score by age, sex, and comorbidity. Cox proportional-hazards regression was used to assess the risk of CVD. Patients with colectomy were found to be at lower risk of CVD (hazard ratio [HR]: 0.95, 95% confidence interval [CI] = 0.90–0.99) than patients without colectomy. Stratified analysis according to the type of surgery revealed patients who underwent cecectomy and right hemicolectomy were at lower risk of CVD (cecectomy: adjusted HR [aHR] = 0.77, 95% CI = 0.64–0.94; right hemicolectomy: aHR = 0.88, 95% CI = 0.82–0.96). Patients who underwent left hemicolectomy were at higher risk of CVD (aHR = 1.19, 95% CI = 1.08–1.32). Our results indicate that the different colectomy procedures influence the risk for the CVD differently.

## Introduction

Cardiovascular disease (CVD) is the leading cause of death in developed countries, and is currently responsible for one third of the total deaths in the United States per year^1^ and 45% of all deaths in Europe^2^. Cardiometabolic disorders such as obesity, type 2 diabetes and metabolic syndrome have been reported to be associated with long-term exposure to air pollution^3^, life style factors^4^, and nutrition^5^. Alterations in the composition of the gut microbiota also affect host metabolism, with a consequent contribution to the occurrence of cardiometabolic disorders^6^.

Colectomy is commonly performed to treat diseases of the colon. There are limited reports about the association between colectomy and the risk of CVD. A Danish group hypothesized that colectomy might result in changes to the microbiota and, consequently, influence the risk of CVD^7^. Their study indicated that total colectomy decreases the risk of hypertension, while another study reported the increased risk of diabetes among patients undergoing left colectomy or total colectomy^8^. These findings indicate that colectomy may be related to physiological or metabolic functions that are associated with an increased risk of CVD.

The colon is not only critical for maintaining salt and water balances, but also represents the most important reservoir of gut microbiota^9^. Recently, the function and microbiota of the colon have been suggested to play a role in cardiometabolic disorders^10^. Furthermore, microbial sequencing has revealed the presence of particular gut microbiota to be associated with CVD, such as genera of Clostridiales or Clostridium sp. SS2/1^11,12^. The gut microbiota is involved in the regulation of multiple metabolic, signaling, and immune-inflammatory pathways related to physiological functions of the gastrointestinal tract, liver, muscle, and brain^13^. Imbalances in the composition of intestinal microbiota and bacterial metabollites^14–17^ are associated with gastrointestinal disorders, CVD, and systemic illness^18^.

We hypothesized that colorectal procedures may have an impact on the risk of CVD by altering the intestinal microbiota. The present population-based study was carried out in Taiwan to evaluate the risk of CVD after colectomy among patients without colorectal cancer. We excluded patients with colon cancer because adjuvant chemotherapy for colon cancer is associated with hypertension and diabetes, which both increase the risk for cardiovascular morbidities^19,20^.

## Methods

### Ethics statement

The Research Ethics Committee of China Medical University and Hospital in Taiwan approved this study (CMUH-104-REC2–115-R3) and the waiver of written informed consent. All personal information was removed from the dataset prior to analysis. This research was performed in accordance with the relevant guidelines and regulations.

### Data source

The Taiwan National Health Insurance program (Taiwan NHI) is a single-payer insurance system which provides universal coverage for approximately 99% of the population of Taiwan and contracts with 97% of the medical providers^21,22^. The National Health Insurance Research Database (NHIRD) has been created based on the Taiwan NHI for the purposes of research^23^. We conducted a search of the nation-wide hospitalization file based on the NHIRD. All diagnoses in the database were coded according to the International Classification of Disease, Ninth Revision, Clinical Modification (ICD-9-CM).

### Study population

We recruited patients who underwent colectomy for treatment of conditions affecting the colon (classified as ICD-9-OP 45.71 − 45.76, 45.79, and 45.8) between 2000 and 2012. The index date of the colectomy group was the date of colectomy. Exclusion criteria were: colorectal cancer, previous CVD, age <20 years, previous diagnosis of colorectal cancer, and diagnosis of CVD within 3 months after the index date. Patients with a history of hospitalization and care by gastroenterologists, general surgeons, or colorectal surgeons were selected as the control group patients. We used 1:1 propensity score matching to match patients without colectomy to those with colectomy according to age, sex, index year, and comorbidities. All patients were monitored from the index date until either the occurrence of CVD, withdrawal from the database, or December 31, 2013.

CVD was defined as a diagnosis of heart disease (ICD-9-CM 402, 410 − 414, or 420 − 429) or cerebrovascular disease (ICD-9-CM 430 − 438) confirmed at two or more outpatient office visits or during one period of hospitalization within the study period.

The following comorbidities were evaluated: hypertension (ICD-9-CM 401 or 405), diabetes mellitus (ICD-9-CM 250), hyperlipidemia (ICD-9-CM 272), obesity (ICD-9-CM 278), chronic obstructive pulmonary disease^24^ (ICD-9-CM 491, 493, or 496); chronic renal disease^25,26^ (ICD-9-CM 582, 583, 585, 586, or 588); liver disease except tumors^27–29^ (ICD-9-CM 571 or 572); anemia^30^ (ICD-9-CM 280–285); and autoimmune disorders^31^. (ICD-9-CM 710 or 714).

### Statistical analysis

Propensity score matching was based on nearest-neighbor matching without replacement using a caliper width within 0.1. The standardized mean difference (SMD) was used to assess differences in each variable between the colectomy and control cohorts; a SMD of <0.1 was considered a negligible difference. The distributions of age, sex, and comorbidities are presented as numbers and percentages. The person-years of follow-up were calculated for each patient based on the time from index date to the diagnosis of CVD, death, or the last follow-up date (December 31, 2013). Hazard ratios (HR) and 95% confidence intervals (95% CI) were estimated using Cox proportional hazard models. The association between colectomy and CVD were analyzed. The cumulative incidence of CVD in the two cohorts was described by Kaplan-Meier plots and tested using the log-rank test. All statistical analyses were performed using SAS statistical software, version 9.4 (SAS Institute Inc., Cary, NC, USA). The Kaplan-Meier plot was plotted with R software. Statistical significance was determined using a two‐tailed test (p < 0.05).

Because the surgical indications for colectomy varied, we analyzed the indications for colectomy and performed the sensitivity test by analyzing patients of the same disease for colectomy. The patients we analyzed in the sensitivity tests were those with diverticula-related disease and benign colorectal tumor. The control group cases we matched in sensitivity tests were those who ever diagnosed with the same disease without colectomy. The same statistical method was applied for sensitivity testing.

## Results

### Demographic characteristics

The demographic characteristics and comorbidities of all patients are shown in Table 1. There were no statistically significant differences in age, sex, or comorbidities between the two cohorts.

**Table 1:** Demographic characteristics and comorbidities of patients who underwent colectomy in Taiwan from 2000 to 2012.

Variable	Total	Non-Colectomy	Colectomy	Standardized mean difference^§^
	N = 36848	n = 18424	n = 18424	
	n	n (%)/mean ± SD	n (%)/mean ± SD	
**Age at baseline**				0.002
<40	6909	3457 (18.8)	3452 (18.7)	
40–64	18181	9083 (49.3)	9098 (49.4)	
≥65	11758	5884 (31.9)	5874 (31.9)	
Mean age^‡^		55.7 (16.4)	55.7 (16.4)	0.003
**Gender**				0.000
Female	16290	8146 (44.2)	8144 (44.2)	
Male	20558	10278 (55.8)	10280 (55.8)	
**Baseline comorbidity**				
Hypertension	4089	2037 (11.1)	2052 (11.1)	0.003
Diabetes mellitus	2818	1405 (7.6)	1413 (7.7)	0.002
Hyperlipidemia	815	396 (2.1)	419 (2.3)	0.008
Obesity	37	17 (0.1)	20 (0.1)	0.005
Pulmonary disease	1298	653 (3.5)	645 (3.5)	0.002
Chronic renal disease	1167	576 (3.1)	591 (3.2)	0.005
Liver disease	2587	1292 (7)	1295 (7)	0.001
Anemia	3330	1663 (9)	1667 (9)	0.001
Autoimmune disease	290	133 (0.7)	157 (0.9)	0.015

Abbreviations: SD, standard deviation.

Key: ^‡^by two-tailed t-test, ^§^a standardized mean difference of <0.1 indicates a negligible difference between the two cohorts.

### Risk of cardiovascular disease in patients with and without colectomy

After adjustment for age, sex, and comorbidities, patients who had undergone colectomy were at lower risk of CVD than those in the control group (HR = 0.95, 95% CI = 0.90 − 0.99, p = 0.05) (Table 2). Age of ≥40 years and male gender were found to have a significant effect on the risk of CVD (both p < 0.001). Hypertension, diabetes mellitus, hyperlipidemia, chronic obstructive pulmonary disease, and chronic renal disease were also associated with increased risk of CVD (all p < 0.001).

**Table 2:** Results of Cox regression analysis of the association of cardiovascular disease with colectomy.

Characteristics	Event	Crude		Adjusted	
	(n = 5404)	HR (95% CI)	p value	HR (95% CI)	p value
**Colectomy**					
No	3144	1(Ref.)		1(Ref.)	
Yes	2260	0.86(0.81–0.91)	<0.001	0.95(0.90–0.99)	0.050
**Age at baseline**					
<40	243	1(Ref.)		1(Ref.)	
40–64	1895	3.89(3.41–4.45)	<0.001	3.67(3.21–4.20)	<0.001
≥65	3266	14.34(12.58–16.35)	<0.001	12.21(10.70–13.94)	<0.001
**Gender**					
Female	2268	1(Ref.)		1(Ref.)	
Male	3136	1.12(1.06–1.18)	<0.001	1.21(1.14–1.27)	<0.001
**Baseline comorbidity**					
Hypertension	1010	2.95(2.75–3.16)	<0.001	1.36(1.26–1.47)	<0.001
Diabetes mellitus	731	2.85(2.64–3.09)	<0.001	1.62(1.49–1.76)	<0.001
Hyperlipidemia	168	1.82(1.56–2.13)	<0.001	1.3(1.11–1.52)	0.001
Obesity	5	1.30(0.54–3.13)	0.553	1.63(0.68–3.94)	0.276
Pulmonary disease	361	2.91(2.61–3.23)	<0.001	1.33(1.19–1.48)	<0.001
Chronic renal disease	325	3.08(2.76–3.45)	<0.001	1.56(1.39–1.76)	<0.001
Liver disease	443	1.53(1.39–1.69)	<0.001	1.21(1.09–1.34)	<0.001
Anemia	579	1.72(1.57–1.87)	<0.001	1.21(1.11–1.33)	<0.001
Autoimmune disease	46	1.43(1.07–1.91)	0.016	1.32(0.98–1.76)	0.063

Abbreviations: HR, hazard ratio; CI, confidence interval; Ref., Reference.

Adjusted HR refers to adjustment for age, sex, and comorbidities in Cox proportional-hazards regression.

**Figure 1:**
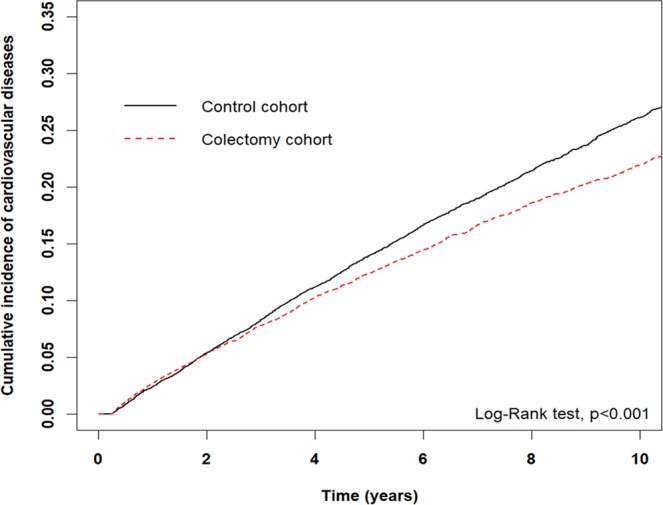
Results of Kaplan-Meier analysis for cumulative incidence of cardiovascular disease. The cumulative incidence of cardiovascular disease was lower in the colectomy than control cohort (p < 0.001).

As Fig. 1 demonstrates, the cumulative incidence of CVD was lower in the colectomy cohort than the control cohort (p < 0.001).

### Cardiovascular risk for different colectomy procedures

Table 3 shows the CVD risk associated with different colectomy procedures. After adjustment for age, sex, and comorbidities, patients who underwent cecectomy (adjusted HR [aHR]: 0.77, 95% CI = 0.64–0.94) and right hemicolectomy (aHR = 0.88, 95% CI = 0.82–0.96) had lower risks for CVD. Left hemicolectomy was associated with a higher risk for CVD (aHR = 1.19, 95% CI = 1.08–1.32).

**Table 3:** Cardiovascular risk according to type of colectomy.

Variable	Event	Person years	IR	Crude HR (95% CI)	Adjusted HR (95% CI)
**Non-colectomy**	3144	105890	29.69	1(Ref.)	1(Ref.)
**Colectomy surgery**					
Cecectomy	107	6642	16.11	0.57(0.47–0.70)***	0.77(0.64–0.94)**
Right hemicolectomy	717	33406	21.46	0.74(0.68–0.80)***	0.88(0.82–0.96)**
Resection of transverse colon	77	2934	26.24	0.95(0.76–1.20)	1.02(0.81–1.28)
Left hemicolectomy	406	10509	38.63	1.43(1.29–1.58)***	1.19(1.08–1.32)***
Sigmoidectomy	471	13568	34.71	1.28(1.16–1.40)***	1.05(0.95–1.15)
Total intra-abdominal colectomy	34	2927	11.62	0.42(0.30–0.58)***	0.82(0.59–1.15)
Partial colectomy, site undetermined	478	20225	23.63	0.84(0.77–0.93)***	0.96(0.88–1.06)

Abbreviations: IR, incidence rate per 1,000 person-years; HR, hazard ratio; CI, confidence interval; Ref., Reference.

Adjusted HR refers to adjustment for sex, age, sex, and comorbidities in Cox proportional-hazards regression.

Key: *p < 0.05; **p < 0.01; ***p < 0.001.

### Cardiovascular risk and different indications for colectomy

Among the case cohort, the top five indications for colectomy were diverticula-related disease (ICD-9-CM 562) in 3,717 (20.2%) patients, benign colorectal tumors (ICD-9-CM 211) in 2,481 (13.5%) patients, bleeding or perforation of the intestine (ICD-9-CM 569) in 1,904 (10.3%) patients, appendicitis (ICD-9-CM 540) in 1,048 (5.7%) patients, intestinal obstruction (ICD-9-CM 560) in 1,031 (5.6%) patients. The top two indications for colectomy were analyzed independently; the leading indication for non-cancer colectomy was diverticula-related disease (Supplementary Table A1), and the second was benign colorectal tumors (Supplementary Table B1).

In Supplementary Table A3, colectomy for diverticula-related disease or right hemicolectomy were associated with a lower risk of CVD (aHR = 0.77, 95% CI = 0.65–0.92) while left hemicolectomy was associated with a higher risk of CVD(aHR = 1.23, 95% CI = 1.01–1.49). The cumulative incidence of CVD was lower among patients who underwent colectomy for diverticular diseases than in the control cohort (p = 0.02, Supplementary Fig. A1). Among patients who underwent colectomy for benign colorectal tumors, right hemicolectomy was associated with a lower risk of CVD (aHR = 0.79, 95% CI = 0.63–0.98, Supplementary Table B3). The cumulative incidence of CVD was lower among patients who underwent colectomy for benign colorectal tumors than in the control cohort (p = 0.004).

## Discussion

The results of this population-based study indicate that cecectomy and right hemicolectomy are associated with a decreased risk of CVD among patients without colorectal cancer. However, left hemicolectomy may be associated with an increased risk of CVD.

We identified that age of ≥40 years and male sex, as well as the presence of comorbidities, are important risk factors for CVD, which supports the results of a previous study^32^. We found colectomy to be a protective factor for CVD; however, when results were stratified according to the type of surgery, the risk of CVD varied dramatically. The protective effect of colectomy was most significant in the case of right hemicolectomy.

Diverticular disease and benign colorectal tumors are the leading two indications for colectomy. Diverticula-related diseases have been reported to increase the risk of CVD^33,34^; in the present study, the rate of CVD was even lower among patients with diverticula-related diseases after receiving right hemicolectomy procedure. This suggests that right hemicolectomy may be a protective factor for CVD, even in the case of diverticula-related disease. Among patients with benign colorectal tumors, right colectomy was also associated with a lower risk of CVD. Our results showed a consistent trend of decreasing CVD risk in patients who had undergone right hemicolectomy.

The colon is responsible for the propulsion of colonic content toward eventual expulsion and the absorption of water, electrolytes, and short-chain fatty acids (SCFA) that are produced by symbiotic bacteria^35^. Bacterial load, degree of fermentation, and proliferation are highest in the proximal colon^36^. Fermented metabolites such as trimethylamine-N-oxide (TMAO) and SCFA contribute to the host-gut microbiota interactions that lead to CVD^37^. Although fermentation of amino acids produces beneficial SCFAs, the process also leads to the production of a range of potentially harmful compounds which may play a role in the development of CVD, colon cancer, and inflammatory bowel disease^37–39^. Our results indicate that different colectomy procedures are associated with different CVD risks. We found removal of the proximal colon by cecectomy and right hemicolectomy to be associated with reduction of CVD risk. Although this somewhat contradicts the results of Jensen’s study, which did not find an association between colectomy and the risk of CVD^7^, the previous study included patients with colon cancer. Adjuvant chemotherapy for colon cancer is associated with hypertension and diabetes, which both increase the risk of cardiovascular morbidity^20^. Physiological function, microbiota composition, and fermentation differ between the left and right colon^36,40^. It is interesting that our data also revealed different risks for CVD in relation to left or right colectomy procedures. Additional research is necessary to evaluate whether these findings can be attributed to differences in the gut microbiota.

The strength of this study is that it is a nation-wide, population-based, cohort design with almost complete follow-up data evaluated using access to healthcare services^41^. Additionally, this study avoided any inherent bias toward identification of increased risk of CVD by excluding patients with colorectal cancer. Patients with colorectal cancer who have received adjuvant chemotherapy have been reported to have 3.07-fold increased risk of CVD^20^.

There are some limitations to this study which should be acknowledged. First, risk factors for CVD such as smoking, diet, and inactivity are not included in NHIRD. Although we were unable to obtain this information, we adjusted for comorbidities that are known risk factors for CVD. After adjustment, proximal colectomy remained a significant protective factor for CVD. Second, inaccurate classification of CVD may have occurred; however, the aim of the study was to compare the overall risk of CVD rather than risks for specific types of CVD^7^.

In conclusion, our results suggest that proximal colectomy and left colectomy are associated with a decreased and increased risk of CVD, respectively. The underlying causes of these opposing effects results require additional research. More physiological studies are necessary to establish the association of colectomy procedures with metabolic alterations and gut dysbiosis. Future studies may include comparisons of gut microbiota and lipid profile before and after different types of colectomy. In clinical application, we may consider to treat colon as an important organ more than maintaining salt, water balances and fecal propulsion.

## Supplementary information

Supplementary tables and figures.

## Data Availability

All data that were generated or analyzed during this study are included in the dataset and can be requested from the Taiwan National Health Institute. Due to restrictions imposed by the government of Taiwan in relation to the “Personal Information Protection Act”, data cannot be made available to the public. Formal requests for data can be sent to the NHIRD (http://nhird.nhri.org.tw).
