# Supplementary material for: The impact of colectomy on the risk of cardiovascular disease among patients without colorectal cancer
Source: Sci Rep. 2020 Feb 19;10:2925. doi: 10.1038/s41598-020-59640-w (PMC7031401; doi:10.1038/s41598-020-59640-w)
Supplement: Supplementary file 1 — Supplementary tables and figures. [file 41598_2020_59640_MOESM1_ESM.docx]

**The impact of colectomy on the risk of cardiovascular disease among patients without colorectal cancer-Supplementary tables and figures**

Chin-Chia Wu^1,6,7^, Ta-Wen Hsu^1,6^, Chia-Chou Yeh^2,7^, Cheng-Hung Lee^3,6,7^, Mei-Chen Lin^4,8^, Chun-Ming Chang^5,6#^

**Key Words:** colectomy; cardiovascular disease; gut microbiota

**Correspondence and requests for materials should be addressed to Chun-Ming Chang, email: ccmjim1008@gmail.com**

| **Supplement A: Colectomy for diverticula-related disease**  **Supplementary Table A1. Demographic characteristics and comorbidities of patients with diverticula-related disease who underwent colectomy in Taiwan from 2000 to 2012** | | | | |
| --- | --- | --- | --- | --- |
|  |  | **Diverticular diseases of colon** | |  |
| **Variable** | **Total** | **Non-Colectomy** | **Colectomy** | **Standardized mean difference§** |
|  | **N=5154** | **n=2912** | **n=2912** |  |
|  | **n** | **n (%) / mean ± SD** | **n (%) / mean ± SD** |  |
| **Age at baseline** |  |  |  | 0.008 |
| <40 | 1260 | 633 (21.7) | 627 (21.5) |  |
| 40-64 | 2939 | 1464 (50.3) | 1475 (50.7) |  |
| ≥65 | 1625 | 815 (28) | 810 (27.8) |  |
| Mean age‡ |  | 53.8 (16.2) | 53.9 (16.0) | 0.001 |
| **Gender** |  |  |  | 0.007 |
| Female | 2248 | 1129 (38.8) | 1119 (38.4) |  |
| Male | 3576 | 1783 (61.2) | 1793 (61.6) |  |
| **Baseline comorbidity** |  |  |  |  |
| Hypertension | 486 | 231 (7.9) | 255 (8.8) | 0.030 |
| Diabetes mellitus | 294 | 131 (4.5) | 163 (5.6) | 0.050 |
| Hyperlipidemia | 134 | 57 (2) | 77 (2.6) | 0.046 |
| Obesity | 0 | 0 (0.0) | 0 (0.0) | 0.000 |
| Pulmonary disease | 186 | 88 (3) | 98 (3.4) | 0.020 |
| Chronic renal disease | 130 | 57 (2) | 73 (2.5) | 0.040 |
| Liver disease | 308 | 147 (5) | 161 (5.5) | 0.021 |
| Anemia | 254 | 125 (4.3) | 129 (4.4) | 0.007 |
| Autoimmune disease | 50 | 20 (0.7) | 30 (1) | 0.037 |
| Abbreviations: SD, standard deviation.  Key: ‡ by two-tailed t-test, § a standardized mean difference of ≤0.1 indicates a negligible difference between groups. | | | | |

| **Supplementary Table A2. Results of Cox regression analysis of the association of cardiovascular disease with colectomy for diverticula-related diseases** | | | | | | |
| --- | --- | --- | --- | --- | --- | --- |
| **Characteristics** | **Event** | **Crude** | |  | **Adjusted** | |
|  | **(n=1040)** | **HR (95% CI)** | **p value** |  | **HR (95% CI)** | **p value** |
| **Colectomy** |  |  |  |  |  |  |
| No | 571 | 1(Ref.) |  |  | 1(Ref.) |  |
| Yes | 469 | 0.86(0.77-0.98) | 0.020 |  | 0.92(0.81-1.04) | 0.161 |
| **Age at baseline** |  |  |  |  |  |  |
| <40 | 38 | 1(Ref.) |  |  | 1(Ref.) |  |
| 40-64 | 400 | 5.41(3.88-7.54) | <0.001 |  | 5.22(3.74-7.28) | <0.001 |
| ≥65 | 602 | 21.9(15.76-30.42) | <0.001 |  | 19.46(13.95-27.15) | <0.001 |
| **Gender** |  |  |  |  |  |  |
| Female | 453 | 1(Ref.) |  |  | 1(Ref.) |  |
| Male | 587 | 0.78(0.69-0.88) | <0.001 |  | 1.11(0.98-1.26) | 0.116 |
| **Baseline comorbidity** |  |  |  |  |  |  |
| Hypertension | 153 | 3.25(2.73-3.86) | <0.001 |  | 1.47(1.21-1.77) | <0.001 |
| Diabetes mellitus | 97 | 2.99(2.43-3.69) | <0.001 |  | 1.49(1.19-1.86) | <0.001 |
| Hyperlipidemia | 35 | 1.88(1.34-2.63) | <0.001 |  | 1.41(0.99-2.02) | 0.058 |
| Obesity | 0 | - | - |  | - | - |
| Pulmonary disease | 71 | 3.04(2.39-3.86) | <0.001 |  | 1.37(1.07-1.76) | 0.013 |
| Chronic renal disease | 52 | 4.63(3.5-6.13) | <0.001 |  | 1.9(1.39-2.59) | <0.001 |
| Liver disease | 73 | 1.56(1.23-1.98) | <0.001 |  | 1.3(1.01-1.67) | 0.038 |
| Anemia | 57 | 2.05(1.57-2.67) | <0.001 |  | 1.14(0.85-1.52) | 0.385 |
| Autoimmune disease | 14 | 2.39(1.41-4.06) | 0.001 |  | 1.97(1.15-3.37) | 0.013 |
| Abbreviations: HR, hazard ratio; CI, confidence interval; Ref., Reference. | | | | | | |
| Adjusted HR: adjusted for age, sex, and comorbidities in Cox proportional-hazards regression. | | | | | | |

| \| **Supplementary Table A3. Cardiovascular risk according to type of colectomy in the patients with diverticula-related disease** \| \| \| \| \| \| \| --- \| --- \| --- \| --- \| --- \| --- \| \| **Variable** \| **Event** \| **Person years** \| **IR** \| **Crude HR (95% CI)** \| **Adjusted HR (95% CI)** \| \| **Non-colectomy** \| 571 \| 18418 \| 31.00 \| 1(Ref.) \| 1(Ref.) \| \| **Colectomy surgery** \|  \|  \|  \|  \|  \| \| Cecectomy \| 20 \| 1743 \| 11.47 \| 0.39(0.25-0.60)*** \| 0.73(0.47-1.14) \| \| Right hemicolectomy \| 152 \| 8657 \| 17.56 \| 0.54(0.45-0.64)*** \| 0.77(0.65-0.92)** \| \| Resection of transverse colon \| 4 \| 163 \| 24.61 \| 0.85(0.32-2.26) \| 1.48(0.55-3.96) \| \| Left hemicolectomy \| 121 \| 2143 \| 56.45 \| 2.08(1.72-2.52)*** \| 1.23(1.01-1.49)* \| \| Sigmoidectomy \| 125 \| 2727 \| 45.84 \| 1.67(1.39-2.02)*** \| 1.04(0.86-1.26) \| \| Total intra-abdominal colectomy \| 2 \| 88 \| 22.82 \| 0.79(0.20-3.16) \| 0.46(0.12-1.87) \| \| Partial colectomy, site undetermined \| 54 \| 2288 \| 23.61 \| 0.81(0.61-1.06) \| 0.93(0.71-1.23) \| \| Abbreviations: IR, incidence rate per 1,000 person-years; HR, hazard ratio; CI, confidence interval; Ref., Reference. \| \| \| \| \| \| \| Adjusted HR: adjusted for sex, age, sex, and comorbidities in Cox proportional-hazards regression. \| \| \| \| \| \| \| * p value<0.05; ** p value<0.01; *** p value<0.001 \| \| \| \| \| \| |
| --- | --- | --- | --- | --- | --- | --- | --- | --- | --- | --- | --- | --- | --- | --- | --- | --- | --- | --- | --- | --- | --- | --- | --- | --- | --- | --- | --- | --- | --- | --- | --- | --- | --- | --- | --- | --- | --- | --- | --- | --- | --- | --- | --- | --- | --- | --- | --- | --- | --- | --- | --- | --- | --- | --- | --- | --- | --- | --- | --- | --- | --- | --- | --- | --- | --- | --- | --- | --- | --- | --- | --- | --- | --- | --- | --- | --- | --- | --- | --- | --- | --- | --- | --- | --- |

|  |
| --- |

**Supplementary Figure A1.** Kaplan-Meier analysis for cumulative incidence of cardiovascular disease. The cumulative incidence of cardiovascular disease was lower among patients with diverticula-related disease who underwent colectomy than in the control cohort (p = 0.02).


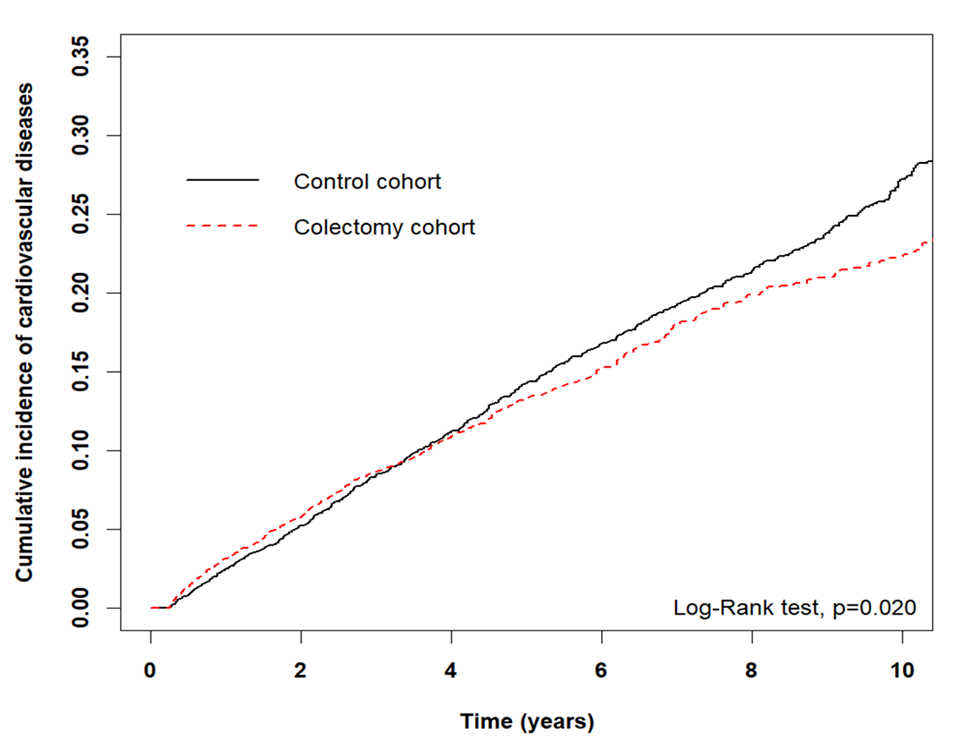


| **Supplement B: Colectomy for benign colorectal tumor**  **Supplementary Table B1. Demographic characteristics and comorbidities of patients with benign colorectal tumor who underwent colectomy in Taiwan from 2000 to 2012** | | | | |
| --- | --- | --- | --- | --- |
|  |  | **Benign colorectal tumor** | |  |
| **Variable** | **Total** | **Non-Colectomy** | **Colectomy** | **Standardized mean difference§** |
|  | **N=4514** | **n=2257** | **n=2257** |  |
|  | **n** | **n (%) / mean ± SD** | **n (%) / mean ± SD** |  |
| **Age at baseline** |  |  |  | 0.009 |
| <40 | 422 | 211 (9.3) | 211 (9.3) |  |
| 40-64 | 2675 | 1333 (59.1) | 1342 (59.5) |  |
| ≥65 | 1417 | 713 (31.6) | 704 (31.2) |  |
| Mean age‡ |  | 58.3 (13.1) | 58.1 (13.0) | 0.015 |
| **Gender** |  |  |  | 0.009 |
| Female | 2038 | 1014 (44.9) | 1024 (45.4) |  |
| Male | 2476 | 1243 (55.1) | 1233 (54.6) |  |
| **Baseline comorbidity** |  |  |  |  |
| Hypertension | 364 | 180 (8) | 184 (8.2) | 0.007 |
| Diabetes mellitus | 196 | 98 (4.3) | 98 (4.3) | 0.000 |
| Hyperlipidemia | 49 | 16 (0.7) | 33 (1.5) | 0.073 |
| Obesity | 5 | 2 (0.1) | 3 (0.1) | 0.013 |
| Pulmonary disease | 97 | 41 (1.8) | 56 (2.5) | 0.046 |
| Chronic renal disease | 34 | 15 (0.7) | 19 (0.8) | 0.020 |
| Liver disease | 157 | 71 (3.1) | 86 (3.8) | 0.036 |
| Anemia | 113 | 48 (2.1) | 65 (2.9) | 0.048 |
| Autoimmune disease | 12 | 3 (0.1) | 9 (0.4) | 0.052 |
| Abbreviation: SD, standard deviation. ‡t-test , §A standardized mean difference of ≤0.1 indicates a negligible difference. Abbreviation: SD, standard deviation. | | | | |

| **Supplementary Table B2. Results of Cox regression analysis of the association of cardiovascular disease with colectomy for benign colorectal tumor** | | | | | | |
| --- | --- | --- | --- | --- | --- | --- |
| **Characteristics** | **Event** | **Crude** | |  | **Adjusted** | |
|  | **(n=566)** | **HR (95% CI)** | **p value** |  | **HR (95% CI)** | **p value** |
| **Colectomy** |  |  |  |  |  |  |
| No | 320 | 1(Ref.) |  |  | 1(Ref.) |  |
| Yes | 246 | 0.78(0.66-0.93) | 0.004 |  | 0.81(0.68-0.95) | 0.012 |
| **Age at baseline** |  |  |  |  |  |  |
| <40 | 18 | 1(Ref.) |  |  | 1(Ref.) |  |
| 40-64 | 226 | 2.99(1.85-4.83) | <0.001 |  | 2.74(1.69-4.44) | <0.001 |
| ≥65 | 322 | 8.81(5.47-14.19) | <0.001 |  | 7.31(4.53-11.82) | <0.001 |
| **Gender** |  |  |  |  |  |  |
| Female | 191 | 1(Ref.) |  |  | 1(Ref.) |  |
| Male | 375 | 1.71(1.44-2.04) | <0.001 |  | 1.59(1.34-1.9) | <0.001 |
| **Baseline comorbidity** |  |  |  |  |  |  |
| Hypertension | 78 | 2.64(2.08-3.36) | <0.001 |  | 1.55(1.2-2) | <0.001 |
| Diabetes mellitus | 49 | 2.78(2.08-3.73) | <0.001 |  | 1.91(1.39-2.6) | <0.001 |
| Hyperlipidemia | 9 | 1.85(0.96-3.57) | 0.068 |  | 1(0.51-1.97) | 0.993 |
| Obesity | 0 | - | - |  | - | - |
| Pulmonary disease | 29 | 3.43(2.36-4.98) | <0.001 |  | 1.92(1.31-2.82) | <0.001 |
| Chronic renal disease | 8 | 1.93(0.96-3.89) | 0.064 |  | 1.24(0.59-2.59) | 0.567 |
| Liver disease | 26 | 1.58(1.06-2.34) | 0.024 |  | 0.99(0.66-1.5) | 0.976 |
| Anemia | 20 | 1.75(1.12-2.74) | 0.014 |  | 1.48(0.93-2.37) | 0.097 |
| Autoimmune disease | 2 | 1.84(0.46-7.39) | 0.388 |  | 1.69(0.42-6.8) | 0.463 |
| *Abbreviation: HR, hazard ratio; CI, confidence interval; Ref., Reference | | | | | | |
| *Adjusted HR: adjusted for age, sex, and comorbidities in Cox proportional hazards regression. | | | | | | |

| \| **Supplementary Table B3. Cardiovascular risk according to type of colectomy the patients with benign colorectal tumor** \| \| \| \| \| \| \| --- \| --- \| --- \| --- \| --- \| --- \| \| **Variable** \| **Event** \| **Person years** \| **IR** \| **Crude HR (95% CI)** \| **Adjusted HR (95% CI)** \| \| **Non-colectomy** \| 320 \| 11483 \| 27.87 \| 1(Ref.) \| 1(Ref.) \| \| **Colectomy surgery** \|  \|  \|  \|  \|  \| \| Cecectomy \| 13 \| 350 \| 37.17 \| 1.46(0.84-2.53) \| 1.36(0.78-2.36) \| \| Right hemicolectomy \| 89 \| 4287 \| 20.76 \| 0.82(0.65-1.03) \| 0.79(0.63-0.98)* \| \| Resection of transverse colon \| 11 \| 532 \| 20.70 \| 0.85(0.47-1.54) \| 0.94(0.52-1.71) \| \| Left hemicolectomy \| 24 \| 1269 \| 18.91 \| 0.76(0.50-1.14) \| 0.80(0.53-1.20) \| \| Sigmoidectomy \| 64 \| 2558 \| 25.02 \| 1.02(0.79-1.32) \| 0.93(0.72-1.21) \| \| Total intra-abdominal colectomy \| 4 \| 423 \| 9.45 \| 0.36(0.13-0.95)* \| 0.93(0.35-2.50) \| \| Partial colectomy, site undetermined \| 44 \| 1986 \| 22.16 \| 0.85(0.63-1.16) \| 0.95(0.70-1.29) \| \| Abbreviation: IR, incidence rates, per 1,000 person-years; HR, hazard ratio; CI, confidence interval; Ref., Reference \| \| \| \| \| \| \| Adjusted HR: adjusted for age, sex, and comorbidities in Cox proportional hazards regression. \| \| \| \| \| \| \| * p value<0.05; ** p value<0.01; *** p value<0.001 \| \| \| \| \| \| |
| --- | --- | --- | --- | --- | --- | --- | --- | --- | --- | --- | --- | --- | --- | --- | --- | --- | --- | --- | --- | --- | --- | --- | --- | --- | --- | --- | --- | --- | --- | --- | --- | --- | --- | --- | --- | --- | --- | --- | --- | --- | --- | --- | --- | --- | --- | --- | --- | --- | --- | --- | --- | --- | --- | --- | --- | --- | --- | --- | --- | --- | --- | --- | --- | --- | --- | --- | --- | --- | --- | --- | --- | --- | --- | --- | --- | --- | --- | --- | --- | --- | --- | --- | --- | --- |

**Supplementary Figure B1.** Kaplan-Meier analysis for cumulative incidence of cardiovascular disease. The cumulative incidence of cardiovascular disease was lower among patients with benign colorectal tumors who underwent colectomy than in the control cohort (p = 0.004).

**
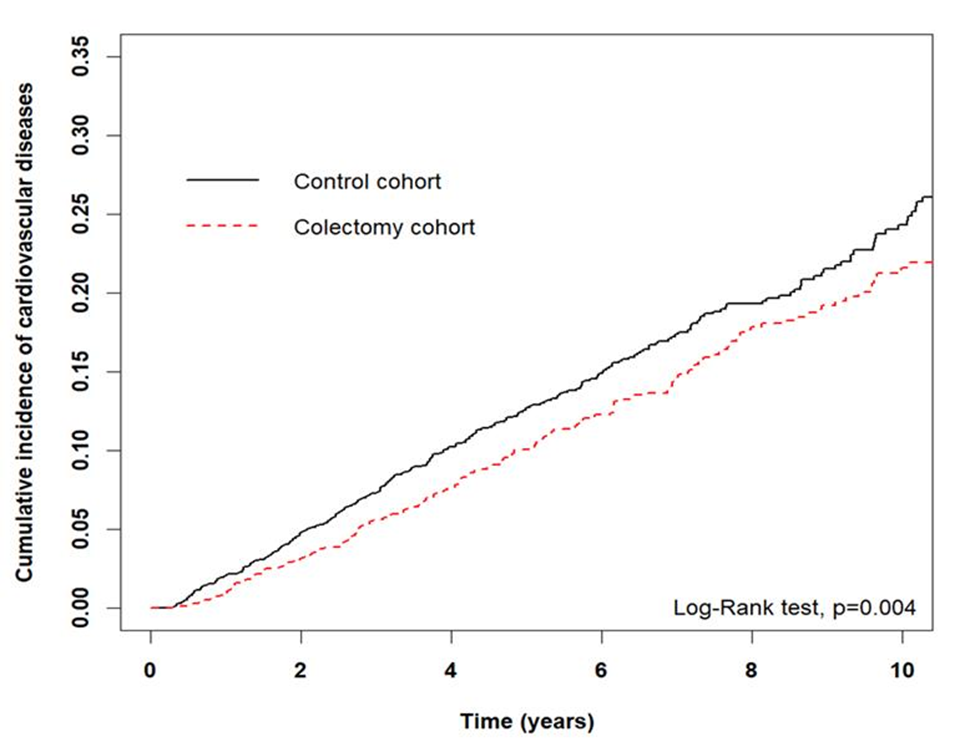
**
